# Dehydrothermally Cross-Linked Collagen Membrane with a Bone Graft Improves Bone Regeneration in a Rat Calvarial Defect Model

**DOI:** 10.3390/ma10080927

**Published:** 2017-08-10

**Authors:** Yin-Zhe An, Young-Ku Heo, Jung-Seok Lee, Ui-Won Jung, Seong-Ho Choi

**Affiliations:** 1 Department of Periodontology, Research Institute for Periodontal Regeneration, College of Dentistry, Yonsei University, Seoul 03722, Korea; yinzhe1226@daum.net (Y.-Z.A.); cooldds@yuhs.ac (J.-S.L.); drjew@yuhs.ac (U.-W.J.); 2 Global Academy of Osseointegration, Neobiotech Co., Ltd., Seoul 06072, Korea; 21cimplant@gmail.com

**Keywords:** allograft, bone regeneration, collagen, cross-linking, dehydrothermal

## Abstract

In this study, the bone regeneration efficacy of dehydrothermally (DHT) cross-linked collagen membrane with or without a bone graft (BG) material was evaluated in a critical-sized rat model. An 8-mm-diameter defect was created in the calvaria of 40 rats, which were randomized into four groups: (1) control; (2) DHT; (3) BG; and, (4) DHT + BG. Evaluations were made at 2 and 8 weeks after surgery using micro-computed tomographic (micro-CT), histological, and histomorphometric analyses. Micro-CT analysis showed an increase in the new bone volume (NBV) of the BG and DHT + BG groups at 2 weeks after surgery, representing a significant difference (*p* < 0.05). At 8 weeks after surgery, the NBV increased in all four groups. However, larger NBVs were observed in the BG and DHT + BG groups, and a significant difference was no longer observed between the two groups. Histologic analysis demonstrated that the graft materials sustained the center of the defect in the BG and DHT + BG groups, which was shown in histomorphometric analysis as well. These results suggest that DHT membrane is a safe biomaterial with adequate tissue integration, and has a positive effect on new bone formation. Moreover, the best effects were achieved when DHT was used in conjunction with BG materials.

## 1. Introduction

The presence of sufficient bone volume is a prerequisite for the predictable osseointegration of a dental implant. However, alveolar ridge resorption over time, combined with tooth loss and the presence of anatomical structures such as the maxillary sinus, nasal cavity, and inferior alveolar nerve all limit the amount of available bone for implant placement [1,2]. Therefore, a bone graft (BG) is often required for the bone regeneration of osseous defects prior to, or simultaneously with implant placement.

Guided bone regeneration (GBR) is a well-established and widely used technique that promotes new bone formation using a barrier membrane to exclude epithelial and connective tissue proliferation within the defect [3,4]. The barrier membranes utilized in the GBR procedure should meet the following requirements: biocompatibility, cell occlusion, host tissue integration, easy manageability, space-maintaining ability during the early stage of healing, and comfort for the patient [5]. Various non-degradable and degradable membranes have been developed according to these requirements, and several non-degradable membranes, such as titanium mesh and expanded polytetrafluoroethylene, showed successful outcomes in clinical and animal studies [6,7,8,9]. Although these membranes are currently considered gold-standard materials, they have a fatal disadvantage. These membranes are susceptible to exposure, resulting in an increased risk of infection and often requiring a second surgery. To avoid a second surgery and to overcome the disadvantages of available membranes, several degradable materials such as collagen, polyglycolide, and polylactic acid have been identified and developed for the use in GBR [4,5,10,11]. However, degradable membranes made from polymers may not be suitable for this purpose because of the presence of non-enzymatic cleavage, leading to acid production with subsequent adverse events [10].

Since collagen is the main component of periodontal tissues, collagen-based materials are representative degradable membranes with many advantageous properties. These include minimal inflammation rates, low immunogenicity and cytotoxicity, hemostasis, and the ease of manipulation during surgical procedures [11]. Collagen can be extracted industrially from bovine and porcine dermis and tendons, and it has multiple applications in periodontal and implant surgeries. Successful GBR, using a non-cross-linked collagen membrane, has been reported [3,4,12]. However, the key disadvantage of such membranes is that they can be quickly resorbed and do not maintain the underlying secluded space for a sufficiently long time to allow the coagulum to appropriately mature and achieve selective repopulation [13]. To prolong the resorption rate, many physical and chemical cross-linking techniques have been applied, such as ultraviolet radiation, dehydrothermal (DHT), glutaraldehyde, and diphenyl-phosphorylation-azide treatment [11,14,15]. However, cross-linking using glutaraldehyde has been reported to induce a cytotoxic effect accompanied by the failure to integrate with the host tissue. Moreover, previous studies have reported that collagen membranes cross-linked using different methods display substantial differences in terms of biodegradation, biocompatibility, and angiogenesis [5,15].

Several investigations have been performed in a rat calvarial defect model to evaluate the effects of various materials on new bone formation. This model is established by forming a bone defect with the diameter of 8 mm in the center of the parietal bone of a rat calvarium using a trephine bur. This defect is regarded as a critical-sized defect, as it does not spontaneously heal during the lifetime of the animal [16]. This model is also relevant for periodontal research, as the physiological remodeling of calvaria is similar to that of the human mandibular bone [17]. However, animal study limitations are that they will only become valid predictors of human response to exposure and treatment if there is a substantial improvement in the scientific methods and systematic review of the animal study-related literature.

It is essential for collagen membranes utilized in GBR to maintain physical integrity long enough to allow bone regeneration. However, there are few available membranes that can completely satisfy all requirements. Therefore, the purpose of the present study was to evaluate bone regeneration efficacy in a critical-sized rat calvarial defect model, using DHT cross-linked collagen membrane with or without BG material.

## 2. Results

### 2.1. Clinical Observations

The post-operative soft tissue healing was uneventful, with no complications (including the membrane and graft material exposure) or other inflammatory reactions observed in any of the rats. At 2 and 8 weeks post-surgery, surgical sites showed the evidence of incision and sutures. After harvesting of the surgical sites, the bone defect areas were clearly surrounded by periosteum and dura mater under visual inspection.

### 2.2. Micro-Computed Tomography (Micro-CT) Findings

**Figure 1:**
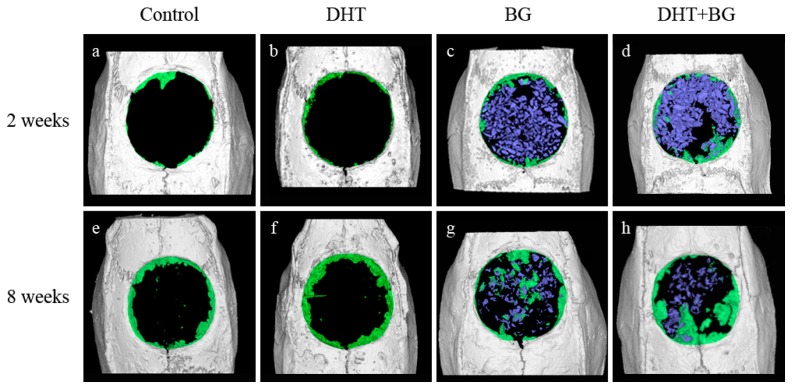
Three-dimensional reconstruction images obtained at 2 and 8 weeks after surgery. (**a,e**) Control group; (**b,f**) DHT membrane group; (**c,g**) BG group; (**d,h**) DHT + BG group. Gray: mother bone; green: new bone; purple: bone graft material.

The results of micro-CT measurements are summarized in Table 1 and Figure 1.

At 2 weeks after surgery, there was no statistically significant difference between the control and DHT groups in terms of total augmented volume (TV), new bone volume (NBV), bone volume fraction (BVF), and bone mineral density (BMD). In contrast to this, TV was significantly increased in both groups receiving BG when compared with that in the control group (BG, *p* = 0.031; DHT + BG, *p* = 0.002). While that of the DHT + BG group was significantly greater than those of the DHT and BG groups (*p* = 0.001 and *p* = 0.016, respectively). Both BG and DHT + BG groups showed significantly higher NBV (all, *p* = 0.008), BVF (vs. control: *p* = 0.009 and 0.008, respectively; vs. DHT group: *p* = 0.008 for both), and BMD (vs. control: *p* = 0.008 and 0.002, respectively; vs. DHT group: *p* = 0.008 for both). Moreover, the NBV and BVF values were significantly higher in the DHT + BG group than in the BG group (*p* = 0.008 and *p* = 0.016, respectively).

At 8 weeks after surgery, all values were shown to have increased, and no statistically significant difference was observed between the control group and DHT group in the four variables. In contrast, the BG and DHT + BG groups showed significantly higher TV values when compared with that in the control group (*p* = 0.008 and *p* < 0.001, respectively). Additionally, the TV value in the DHT + BG group was also significantly higher than those in the DHT and BG groups (*p* = 0.009 and *p* = 0.008, respectively). Similar to the results obtained at 2 weeks, both BG and DHT + BG groups showed significantly higher NBV values (vs. control: *p* = 0.008 and 0.008, respectively; vs. DHT group: *p* = 0.003 and *p* < 0.001, respectively), BVF (vs. control: *p* = 0.002 and *p* < 0.001, respectively; vs. DHT: *p* = 0.003 and *p* < 0.001, respectively), and BMD (vs. control: *p* = 0.004 and *p* < 0.001, respectively; vs. DHT group: *p* = 0.003 and *p* < 0.001, respectively) values. However, no statistically significant difference was observed in NBV, BVF, and BMD values between the BG and DHT + BG groups.

Comparisons within the same experimental groups showed only statistically significant differences in NBV and BVF values in the BG and DHT + BG groups with time (*p* = 0.043 and *p* = 0.043, respectively).

### 2.3. Histological Findings

**Figure 2:**
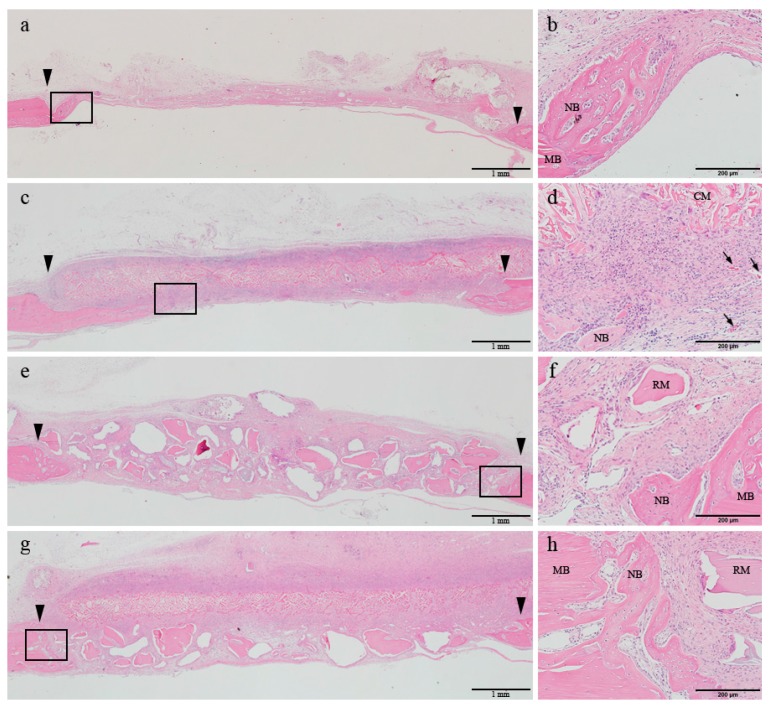
Histologic transversal sections obtained at 2 weeks after surgery (hematoxylin and eosin staining). (**a**,**b**) Control group; (**c**,**d**) DHT membrane group; (**e**,**f**) BG group; (**g**,**h**) DHT + BG group. The boxed areas in the left panels (40× magnification) are magnified in the corresponding panels on the right (200×). Arrowhead: defect margin. MB: mother bone; NB: new bone; CM: collagen membrane; RM: residual material; black arrow: vascular endothelial cells.

At 2 weeks of healing, the early phase of newly formed bone was observed at the margin of the defect in all four groups. In the control and DHT groups, the center of the defect was flattened and mainly occupied by the connective tissue or the grafted collagen membrane. In contrast, in the two groups receiving BG, the center of the defect was sustained by the bone materials (Figure 2a,c,e,g). Under high magnification, a normal post-operative inflammatory response was observed in all four groups. Mononuclear cell infiltration rate in the connective tissue was shown to be pronounced, which was followed by the infiltration of macrophages and lymphocytes. In all four groups, osteoblast and osteoclasts were found to be in close contact with the newly formed bone of the margin of defect. In this period, in the DHT and DHT + BG groups, the membrane was shown to integrate with the surrounding connective tissue and its resorption started. However, the appearance of the membrane body could be easily distinguished from the connective tissue, and the formation of vascular endothelial cells could be observed beneath the membrane. In the BG and DHT + BG groups, multinucleated giant cells were observed around the grafted bone materials (Figure 2b,d,f,h).

**Figure 3:**
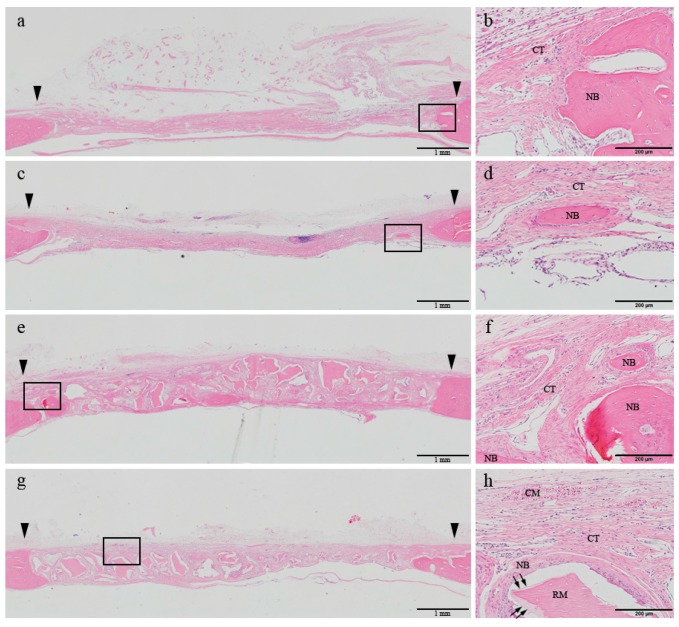
Histologic transversal sections obtained at 8 weeks after surgery (hematoxylin and eosin staining). (**a**,**b**) Control group; (**c**,**d**) DHT membrane group; (**e**,**f**) BG group; (**g**,**h**) DHT + BG group. The boxed areas in the left panels (40× magnification) are magnified in the corresponding panels on the right (200×). Arrowhead: defect margin. CT: connective tissue; NB (black arrow): new bone; CM: collagen membrane; RM: residual material.

At 8 weeks, the woven bone formed in the early phase was shown to be matured, and most of the newly formed bone was apparently regenerated through a centripetal extension from the defect margin in all four groups. In the control group, the center of the defect was depressed and loose connective tissue was shown to be transformed into a well-arranged bundle of fibers. In the DHT group, the collagen membrane body was almost completely resorbed and replaced by newly formed connective tissue. Additionally, a sparser distribution of inflammatory cells could be observed, accompanied by a reduction in their numbers in comparison with those detected at 2 weeks. In a few specimens belonging to the control and DHT groups, bony islands were found in the defect area. In the BG and DHT + BG groups, the defect was well maintained by the allograft materials. Although the number of grafted bone particles was reduced in comparison with those detected at 2 weeks after the surgery, the particles were not completely replaced by the newly formed bone. Bone regeneration was slightly more activated both through the centripetal extension from the margin of the defect, and by formation on the superficial layer of the grafted bone particles (Figure 3).

### 2.4. Histomorphometric Analysis

The results of histomorphometric analysis are summarized in Table 2.

At 2 weeks of healing, no significant differences in TA and NB were observed between the control and DHT groups. In contrast, these values were significantly higher in the BG and DHT + BG groups when compared with those determined in the control group (all *p* < 0.001) and DHT group (*p* = 0.003 and *p* < 0.001, respectively). However, a significant difference was obtained between two BG groups (*p* = 0.009). 

At 8 weeks after surgery, no statistically significant differences were observed in TA and NB values between the control and DHT groups. However, the TA (*p* = 0.008 and *p* < 0.001) and NB (*p* = 0.001 and *p* < 0.001) values in the BG and DHT + BG groups, respectively, were significantly higher when compared with those determined in the control group. The TA values in two BG groups were shown to be significantly different (*p* = 0.009). The NB values were significantly higher in the BG (both *p* < 0.001) and DHT + BG (*p* = 0.001 and 0.003) groups, when compared with those in the control and DHT groups, respectively. TA values were significantly higher in both BG and DHT + BG groups compared with that of determined in the DHT group (*p* = 0.004 and *p* = 0.009, respectively).

In the BG and DHT + BG groups, significant differences in the NB values were detected between 2-week and 8-week healing periods (*p* = 0.043 and *p* = 0.043, respectively). The amount of graft materials, including the bone material and membrane, decreased in all three experimental groups between 2 and 8 weeks after surgery.

### 2.5. Immunohistochemical Findings

**Figure 4:**
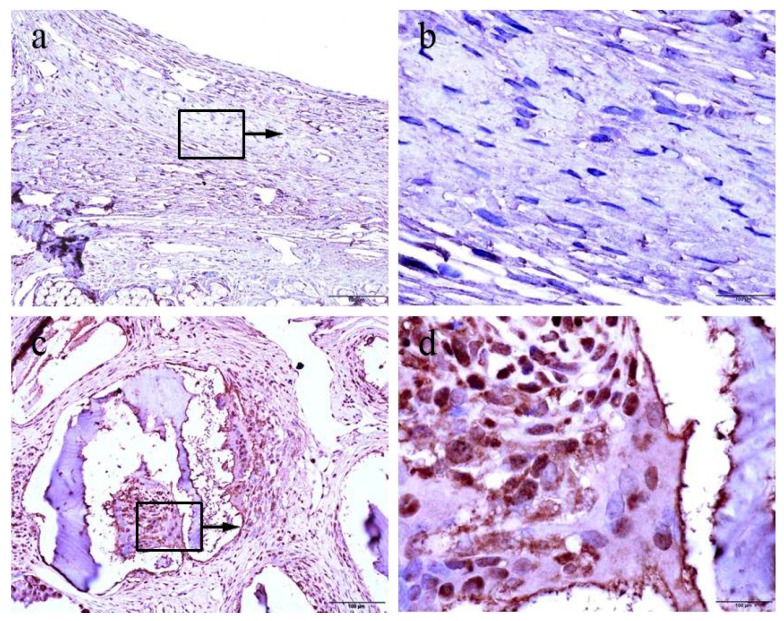
Expression patterns of proliferating cell nuclear antigen (PCNA) in tissue sections detected by immunohistochemistry: example of a negative (**a**,**b**) and positive (**c**,**d**) patterns of PCNA expression in tissue sections. The boxed areas in the left panels (200× magnification) are magnified in the corresponding panels on the right (1000× magnification).

Proliferating cells were highlighted using the proliferating cell nuclear antigen (PCNA) antibody by immunohistochemistry both in the control and the experimental group. We found that the control group mainly showed negative patterns for PCNA expression (Figure 4a,b). By contrast, positive patterns of PCNA expression were frequently detected in the each experimental group (Figure 4c,d).

## 3. Discussion

Biologically, an optimal barrier membrane must induce hemostasis, be integrated by the host tissues, maintain chemotaxis for periodontal ligament fibroblasts, and possess low cytotoxicity. Moreover, the membrane should be able to exclude unwanted cells to protect the wound area and prevent infection [3,4,11]. Therefore, a barrier membrane must be able to maintain its structural integrity during the early healing period. From the clinical point of view, easy manageability and cost-effectiveness are also of great concern when developing a new material. Therefore, many studies focused on these two aspects to develop barrier membranes. Porcine skin-derived collagen membranes are widely used in GBR because their 3D structure is similar to that of the native extracellular matrix. Moreover, the DHT cross-linking technique in porcine collagen membrane may promote mechanical properties [18]. In the present study, a porcine skin-derived type collagen membrane was evaluated using a rat calvarial defect model. The DHT collagen membrane has a positive effect on new bone formation, confirming the results of previous studies [15,19]. Additionally, the DHT membrane displayed excellent integration into the surrounding connective tissue during the early healing period with minimal immune reactions, as histological analysis has demonstrated. Specifically, the histomorphometric analysis showed that the new bone was formed in all four groups, at 2 and 8 weeks after surgery. However, no statistically significant differences were observed between the control and DHT groups. In contrast to this, significantly greater NB values were detected in the BG and DHT + BG groups, when compared with those in the control and DHT groups. Additionally, the differences in NB were significant between the BG and DHT + BG groups. Micro-CT analysis yielded similar results, and the bone of better quality was shown to be produced in the groups containing BG materials. Notably, these results demonstrated that the highest level of new bone formation occurred when the DHT collagen membrane was used in combination with BG materials. Therefore, the DHT membrane has demonstrated it may be a suitable barrier membrane for GBR.

Good biocompatibility and tissue integration are crucial elements for the reduction of the inflammatory response and membrane exposure [11]. These properties are closely related to the cross-linking agents used, which can induce foreign body reactions. The cross-linking of collagen was shown to be associated with decreased tissue integration and angiogenesis, and a slower resorption rate [5]. Tai et al. [20] reported that a cross-linked collagen membrane was more resistant than a non-cross-linked collagen membrane, with more adverse events and a lower rate of new bone regeneration in a human study. However, certain cross-linked techniques were shown to be suitable despite some membrane exposure [3]. Although the early membrane exposure to the oral environment was reported to cause disintegration, the capacity for resistance to proteolysis of a cross-linked membrane was found to be much stronger than that of a non-cross-linked membrane [21]. Unlike materials reported in previous studies, the DHT cross-linked collagen membrane did not induce soft tissue dehiscence at any surgical cites. Furthermore, histological evidence suggested that the DHT membrane was well integrated with the surrounding connective tissues at the early healing stage (i.e., at 2 weeks after surgery). It also had the ability to form vascular endothelial cells, which play an important role during the early new bone formation. These results indicate that the DHT cross-linking technique is a suitable method for achieving a balance between membrane stability and functional remodeling [22].

Various graft materials have been developed and used to obtain favorable outcomes in periodontal and implant surgeries, including autogenous bones, allografts, xenografts, and bone substitutes [23]. Freeze-dried bone allograft (FDBA) may represent a good substitute for autogenous bone. In the present study, FDBA was used to fill the bone defect, which showed a positive effect on the new bone formation. Both BG and DHT + BG groups exhibited a significant increase in the NBV when compared with those in the control and DHT groups at 8 weeks after surgery. In a previous study, various BG materials in conjunction with a titanium membrane were evaluated in a rabbit calvarial defect model at 8 and 16 weeks post-surgery. This demonstrated that the most of newly formed bone was observed even at a relatively early stage (at 8 weeks), while a considerable amount of new bone was formed in the FDBA group at 16 weeks [24], supporting the results of this study. Here, newly formed bone was observed to be in close contact with the FDBA particles. FDBA particles may play an osteoconductive role and serve as the core of new bone deposition. This is supported by the results obtained by Froum et al. and Kolerman et al. Moreover, Piatelli et al. observed the presence of osteoclasts actively resorbing the bone on the outer surface of particles located far from the mother bone [25,26,27]. Collectively, these results show that the particles are resorbed, allowing the deposition of the new bone.

The aim of this study was to evaluate new bone formation using DHT cross-linked collagen membrane with or without bone materials. Some studies reported unfavorable mechanical strength and inadequate barrier function as the major disadvantages of the use of collagen membrane [28,29]. Appropriate space was shown to be obtained when the bone defect morphology is superior, and if the defect cannot be sustained, the membrane itself will inevitably become depressed [30]. Here, the histological evidence revealed that the central portion was depressed or flattened by surrounding connective tissue in the DHT membrane group. Therefore, the results of the present study show that when performing GBR with a collagen membrane, supporting the space with the use of graft material is essential for bone regeneration.

Previous studies reported that a cross-linked collagen membrane promotes new bone formation in bone defects. Park et al. [15] histologically and histomorphometrically evaluated the efficiency of a 1-ethyl-3-(3-dimethylaminopropyl) carbodiimide cross-linked collagen membrane in a rabbit calvarial defect model. Chung et al. [19] clinically and radiographically assessed the bone regeneration capacity of two collagen membranes in human periodontal defects. Although histological analysis and histomorphometric analysis are considered the gold standard in evaluating bone regeneration, a significant correlation between micro-CT and histological analysis has been reported in previous studies [31,32]. In the present study, both histological and histomorphometric analyses were performed to ascertain the effectiveness of the DHT cross-linked collagen membrane for GBR. The results were further corroborated by the results of micro-CT analysis and 3D reconstruction. Micro-CT and a reconstruction program have the advantage of quantifying the parameters accurately, which can serve as guidelines while applying GBR in large animal experiments or human studies [23]. 

According to the results of micro-CT analysis, TV values in the regions of interest increased in a time-dependent manner in all groups. Significant differences in the NBV, BVF, and BMD values, which are the key indices of bone regeneration, were observed between the BG and DHT + BG groups and the control group. Additionally, significant differences were observed between the BG and DHT + BG groups. Based on these results, DHT cross-linked collagen membrane represents a safe biomaterial and has a positive effect on new bone formation, showing good potential to serve as a barrier membrane in the GBR procedure. Given the short evaluation period and the limitations of this study, further investigations are necessary to confirm the effectiveness of the DHT membrane for the GBR in clinical practice.

## 4. Materials and Methods

### 4.1. Experimental DHT Collagen Membrane

The DHT collagen membrane was obtained from the native porcine dermis with additional physical cross-linking, and was mainly composed of type I collagen. The experimental membrane was processed by mechanical cleaning, chemical treatment, lyophilization, and compression. This was followed by the cross-linking using DHT in a vacuum at 110 °C and sterilization. Various DHT membrane properties were investigated, including morphology, enzyme resistance, and mechanical properties. The surface of the DHT membrane had macro-micro interconnective porous structure to permit cellular invasion.

### 4.2. BG Material

FDBA was used as the graft material in this study. The allograft (Regenoss, Cellumed, Seoul, Korea), a particle-type graft material, was composed of a cortical and cancellous powder with a weight ratio of 80/20. The sizes of the particle and pore were 0.2–1.0 mm and 250 µm, respectively.

### 4.3. Experimental Animals

A total of 40 albino rats of the Wistar strain (male, 11 ± 0.5 weeks old) weighing between 350 g and 370 g were used in the present study. All rats were housed individually in standard cages under specific pathogen-free conditions, and fed a standard laboratory diet and water. The animals were allowed to acclimate to the new environment for 7 days before performing the surgery. The animal selection, management, preparation, and surgical protocol were evaluated and approved by the institutional Animal Care and Use Committee, Yonsei Medical Center, Seoul, Korea (approval number 2014-0332).

### 4.4. Study Design

A critical-sized defect of 8 mm in diameter was created in the parietal bone of each rat calvarium. A total of 40 defects were formed. Ten rats were allocated to each of the following groups, which were split into two subgroups for the examination at 2 and 8 weeks after surgery: (1) sham surgery control group, in which the bone discs were removed and the surgical defects were not filled with any membrane or material; (2) DHT membrane group, in which the surgical defects were covered by DHT collagen membranes; (3) BG group, in which the surgical defects were filled with FDBA; and, (4) DHT + BG group, in which the surgical defects were filled with FDBA and covered with DHT collagen membranes.

### 4.5. Surgical Procedure

Initially, the animals were sedated in a chamber with 4% isoflurane (Ifran, Hana Pharm, Kyonggi-Do, Korea) in 100% O_2_ and then anesthetized by an intraperitoneal injection of 15 mg/kg zoletile (Zoletil 50, Virbac, Carros, France) and 10 mg/kg rompun (Bayer, Ansan, Gyeonggi-do, Korea). Under local anesthesia with 2% lidocaine hydrochloride containing 1:100,000 epinephrine, after disinfection with povidone iodine (Povidin, Firson, Cheonan, Chungcheongnam-do, Korea), a middle skin incision was made on the skull, and a full-thickness flap was reflected. Under copious saline irrigation, a standardized, round transosseous defect of 8 mm in diameter was created in the center of the calvaria with a trephine bur. After removal of the trephined calvarial disc, four groups of ten animals each received the DHT collagen membrane, BG, BG with collagen membrane, or sham-surgery control procedure. For the groups with the collagen membrane, it was cut to a size of 10 × 10 mm^2^ squares to cover the outer surface of the bony defect, and then placed over the defect. For the groups involving BG, a sufficient amount of graft material was applied to completely fill the defect by applying a gentle pressure using a surgical instrument. After obtaining adequate hemostasis, the periosteum and skin were repositioned and sutured. Post-operatively, to minimize post-operative pain and prevent infection, all rats were injected subcutaneously with antibiotics (10 mg/day enfloxacin, once daily for 5 days) and analgesics (1 mg/kg meloxicam, once daily for 5 days).

After healing periods of 2 and 8 weeks, five rats in each group were euthanized in a CO_2_ chamber. Subsequently, block sections of the rat calvaria were harvested and fixed in a 10% neutral buffered formalin solution.

### 4.6. Micro-CT Analysis

All samples were scanned using high-resolution micro-CT system (SkyScan 1173, Kontich, Belgium) at a pixel size of 14.91 µm. Prior to scanning the samples, calibration was performed using water, air, and synthetic bone samples. The digital images were obtained under a source voltage of 130 kV and a current of 60 µA. The scanned images were then reconstructed using CT-analyzer software (Ondemand 3D, Cybermed Inc., version 1.0, Seoul, Korea). The regions of interest of each sample were determined in 3D images for the analysis of TV, NBV, BVF (calculated as the NBV divided by TV), and BMD.

### 4.7. Histological and Histomorphometric Analysis

After obtaining micro-CT scans, block sections of the experimental sites were fixed in a 10% neutral-buffered formalin solution for 10 days. The fixed specimens were decalcified in 5% formic acid for 14 days and embedded in paraffin. Serial sections of 5 µm were cut through the central portion of each experimental site. Only the central sections were chosen and stained with hematoxylin-eosin for histological and histomorphometric analysis.

An experienced researcher, blinded to the specific experimental conditions, performed the microscopic examination and histomorphometric analysis. Digital images of histologic slides were obtained using a binocular microscope (Leica DM LB, Leica Microsystems, Wetzlar, Germany), coupled with a color camera (Leica DC300F, Leica Microsystems, Wetzlar, Germany), and saved as digital files. Histometric measurements in the defects were made using automated image analysis software (Image-Pro Plus, Media Cybernetics, Silver Spring, MD, USA) at 12.5× magnification. The following parameters were measured: (1) TA, including newly formed bone, connective tissue, remaining membrane, and grafted materials within the defect; (2) NB, representing the area of newly formed bone within the defect; (3) remaining membrane area (RMA), representing the area of residual membrane within the defect; and (4) residual materials (RM), grafted bone materials within the defect.

### 4.8. Immunohistochemical Analysis

Activity of cell proliferation in new bone area was identified using immunohistochemical analysis with an anti-PCNA monoclonal antibody. Endogenous peroxidase activity was inactivated with 1% hydrogen peroxide solution for 30 min. The sections were blocked with PBS containing 5% bovine serum albumin at room temperature for 10 min and reacted with the primary antibody (1:100, ab29, Abcam, Cambridge, UK) at room temperature for 1 h. After washing in tris-buffered saline, the sections were reacted with secondary antibody (Vectastain ABC kit, Vector laboratories, Burlingame, CA, USA) at room temperature for 30 min. Then, the bound antibodies were visualized with avidin-biotin DAB system (Dako, Glostrup, Denmark), and counter-stained with hematoxylin. The PCNA-positive cells were observed under optical microscopy (Olympus, Tokyo, Japan).

### 4.9. Statistical Analysis

The statistical analysis was performed using a commercially available software program (SPSS 20.0, SPSS Inc., Chicago, IL, USA). The bone defect of each rat was regarded as a statistical unit. Data obtained in each group are expressed as mean values and standard deviations. Kruskal–Wallis one-way analysis of variance based on ranks and the post-hoc Mann–Whitney U test were used to assess the differences among groups at each time point. The Wilcoxon signed rank test was used to compare the data obtained in the same group between two healing periods. A *p*-value less than 0.05 was considered statistically significant.

## Figures and Tables

**Table 1:** Micro-CT measurements of the rat calvarial defect at 2 and 8 weeks after surgery.

	Group	TV (mm^3^)	NBV (mm^3^)	BVF (%)	BMD (mg·mL^−1^)
2 weeks	Control	75.17 ± 3.43	1.43 ± 0.67	1.90 ± 1.28	0.29 ± 0.09
	DHT	87.26 ± 9.18	1.05 ± 0.36	1.20 ± 0.37	0.36 ± 0.14
	BG	97.58 ± 13.93 ^a^	10.48 ± 2.48 ^a,b^	10.74 ± 1.76 ^a,b^	1.92 ± 0.48 ^a,b^
	DHT + BG	122.35 ± 13.05 ^a–c^	16.41 ± 1.99 ^a–c^	13.42 ± 0.86 ^a–c^	2.58 ± 0.49 ^a,b^
8 weeks	Control	85.76 ± 8.12	2.18 ± 1.04	2.52 ± 1.12	0.50 ± 0.13
	DHT	98.78 ± 6.76	3.21 ± 1.10	3.04 ± 1.19	0.68 ± 0.39
	BG	110.02 ± 6.06 ^a^	21.38 ± 6.38 ^a,b,d^	19.38 ± 5.48 ^a,b,d^	3.28 ± 0.99 ^a,b^
	DHT + BG	137.40 ± 3.66 ^a–c^	26.77 ± 2.11 ^a,b,d^	19.52 ± 1.99 ^a,b,d^	4.25 ± 0.48 ^a,b^

TV: total tissue volume; NBV: new bone volume; BVF: bone volume fraction; BMD: bone mineral density. ^a^ Statistically Significant difference from the control group; ^b^ Statistically significant difference from the DHT group; ^c^ Statistically significant difference from the BG group; ^d^ Statistically significant difference from the same experimental group at 2 weeks.

**Table 2:** Histomorphometric measurements at 2 and 8 weeks after surgery.

	Group	Total Augmented Area (TA)	New Bone Area (NB)	Residual Materials	Remaining Membrane
2 weeks	Control	2.71 ± 0.36	0.11 ± 0.08	-	-
	DHT	4.85 ± 0.83	0.20 ± 0.06	-	1.63 ± 0.44
	BG	7.04 ± 1.09 ^a,b^	0.62 ± 0.07 ^a,b^	3.30 ± 0.69	-
	DHT + BG	8.46 ± 0.93 ^a–c^	0.72 ± 0.07 ^a,b^	2.79 ± 0.53	2.16 ± 0.27
8 weeks	Control	4.15 ± 0.73	0.24 ± 0.13	-	-
	DHT	8.09 ± 0.63	0.39 ± 0.08	-	0.27 ± 0.18
	BG	9.91 ± 0.66 ^a–c^	1.35 ± 0.13 ^a,b,d^	1.64 ± 0.88	-
	DHT + BG	14.45 ± 0.57 ^a–c^	1.52 ± 0.38 ^a,b,d^	1.52 ± 0.45	0.03 ± 0.02

TA: total augmented area; NB: new bone area. ^a^ Statistically significant difference from the control group; ^b^ Statistically significant difference from the DHT group; ^c^ Statistically significant difference from the BG group; ^d^ Statistically significant difference from the same experimental group at 2 weeks.
